# Proteolytically Inactive Insulin-Degrading Enzyme Inhibits Amyloid Formation Yielding Non-Neurotoxic Aβ Peptide Aggregates

**DOI:** 10.1371/journal.pone.0059113

**Published:** 2013-04-11

**Authors:** Matias B. de Tullio, Valeria Castelletto, Ian W. Hamley, Pamela V. Martino Adami, Laura Morelli, Eduardo M. Castaño

**Affiliations:** 1 Fundación Instituto Leloir and Instituto de Investigaciones Bioquímicas de Buenos Aires, Consejo Nacional de Investigaciones Científicas y Técnicas (CONICET), Buenos Aires, Argentina; 2 Department of Chemistry, University of Reading, Whiteknights, Reading, United Kingdom; Omaha Veterans Affairs Medical Center, United States of America

## Abstract

Insulin-degrading enzyme (IDE) is a neutral Zn^2+^ peptidase that degrades short peptides based on substrate conformation, size and charge. Some of these substrates, including amyloid β (Aβ) are capable of self-assembling into cytotoxic oligomers. Based on IDE recognition mechanism and our previous report of the formation of a stable complex between IDE and intact Aβ *in vitro* and *in vivo*, we analyzed the possibility of a chaperone-like function of IDE. A proteolytically inactive recombinant IDE with Glu111 replaced by Gln (IDEQ) was used. IDEQ blocked the amyloidogenic pathway of Aβ yielding non-fibrillar structures as assessed by electron microscopy. Measurements of the kinetics of Aβ aggregation by light scattering showed that 1) IDEQ effect was promoted by ATP independent of its hydrolysis, 2) end products of Aβ-IDEQ co-incubation were incapable of “seeding” the assembly of monomeric Aβ and 3) IDEQ was ineffective in reversing Aβ aggregation. Moreover, Aβ aggregates formed in the presence of IDEQ were non-neurotoxic. IDEQ had no conformational effects upon insulin (a non-amyloidogenic protein under physiological conditions) and did not disturb insulin receptor activation in cultured cells. Our results suggest that IDE has a chaperone-like activity upon amyloid-forming peptides. It remains to be explored whether other highly conserved metallopeptidases have a dual protease-chaperone function to prevent the formation of toxic peptide oligomers from bacteria to mammals.

## Introduction

The formation of amyloid is a process by which disordered peptides or specific regions of partially unfolded proteins self-assemble to yield fibrils with a typical morphology, a cross-β structure and affinity to dyes such as Congo red. A growing number of proteins capable of forming amyloid are associated with human pathology, including high prevalence diseases such as type 2 diabetes mellitus (DM2) and dementia. In vitro, amyloid fibril formation follows a typical nucleation-dependent kinetics [1] that includes key variables such as critical concentration, nuclei formation, lag phase, exponential growth and steady state. One or more pathways may lead to the conversion of monomeric soluble proteins or peptides into amyloid fibrils [2], [3], [4]. Alternatively, “off-pathway” oligomeric products relative to amyloid formation may be formed after the initiation of the self-assembly process. This has been shown by using compounds that inhibit amyloid β (Aβ) oligomer formation without affecting fibrillogenesis [5], [6].

Recent work has raised the possibility that fibril formation may be protective to cells by stabilizing non-amyloid cytotoxic intermediates, as proposed for several neurodegenerative diseases [7], [8], [9]. Furthermore, amyloid fibrils may play a physiological role serving as a scaffold for the fast fibrillization of the Mα protein within melanosomes [10]. Yet, the existence of cytotoxic oligomers and protofibrils *in vivo* implies that at least one pathway (either “on or off” relative to fibril formation) of proteins/peptides aggregation is likely a pathological process [9], [11], [12].

Aβ, both in fibrillar and non-fibrillar forms, massively accumulates in the brain in sporadic and hereditary Alzheimer's disease (AD) and amyloid angiopathies [13], [14], [15]. Aβ aggregates are capable of inducing dysfunction or death on neural, glial and vascular cells. Moreover, several coding missense mutations within or around the Aβ sequence in its precursor protein, APP, are associated with familial forms of these diseases [16], [17], [18], [19].

At the subcellular level, Aβ generation has been tracked along the late secretory and endocytic pathways, consistent with the localization of the so called β and γ secretases, major protease complexes that release Aβ peptides from APP [20], [21], [22]. Under normal conditions, cells shed soluble Aβ species to the extracellular compartment without membrane damage [23], yet the sites of their initial oligomerization under pathological conditions remain elusive. Although the bulk of Aβ fibrils in the brain seem to be extracellular, recent studies support that Aβ oligomers may be formed intracellularly [24], [25], [26]. Since self-assembly of Aβ is dependent on monomer concentration and time, the overall rates of its production and removal in a given cellular compartment are key factors in determining the initiation of the amyloidogenic pathway(s).

Insulin-degrading enzyme (IDE), neprilysin (NEP) and endothelin-converting enzyme (ECE) are among the major Aβ proteases with physiological relevance, as shown by gene knock-out and transgenic animal models [27], [28], [29]. Alternatively, the aggregation of Aβ *in vivo* may be modulated by its interaction with molecular chaperones. Studies in cell cultures, transgenic *C. elegans* and *in vitro* biophysical data, support that heat-shock proteins (Hsp) 70–90 and small heat shock proteins (sHsp) with chaperone-like activity interact with Aβ and are capable of reducing fibril formation [30], [31]. In addition to these canonical chaperones, Aβ may interact extracellularly with other proteins with chaperone-like activity. Three secreted glycoproteins, namely, clusterin, haptoglobin and α_2_-macroglobulin (α_2_M), are known to have ATP-independent chaperone-like activity *in vitro* [32], [33], [34], [35], [36]. Among these, the highly stable interaction of Aβ with clusterin, a multi-funcional protein, leads to diverse effects, depending on molar ratios that range from prevention of aggregation to enhancement of fibril formation. Interestingly, HSPs, sHSPs and clusterin expression is increased in AD brains and co-localize with Aβ deposits in neuropil and vessels or in surrounding astrocytes, supporting that they participate in some way in the amyloidogenic process *in vivo* [37], [38], [39], [40].

IDE is a highly conserved Zn^2+^ metallopeptidase ubiquitously expressed in tissues and at the subcellular level. IDE hydrolyzes insulin and Aβ *in vivo* [41] as well as short bioactive peptides with amyloidogenic potential such as glucagon, atrial natriuretic peptide and amylin (reviewed in [42]). We have recently shown that natively folded IDE is capable of forming a highly stable complex (IDE-AβSCx) with Aβ *in vitro* and *in vivo*. The shortest competent Aβ fragment for IDE-AβSCx formation contained the central LVFF sequence, which seems to be crucial for amyloid formation [43], [44], [45]. Interestingly, this sequence is included in the Aβ segment that acquires β-conformation inside the IDE chamber close to the active site [46].

In this study we present results supporting that the catalytically inactive mutant IDEQ drastically modifies the Aβ self-assembly pathway, halts amyloid fibril formation and yields non-toxic aggregates through a chaperone-like activity.

## Results

### Native IDEQ is highly efficient in forming a stable complex with Aβ1-42

**Figure 1:**
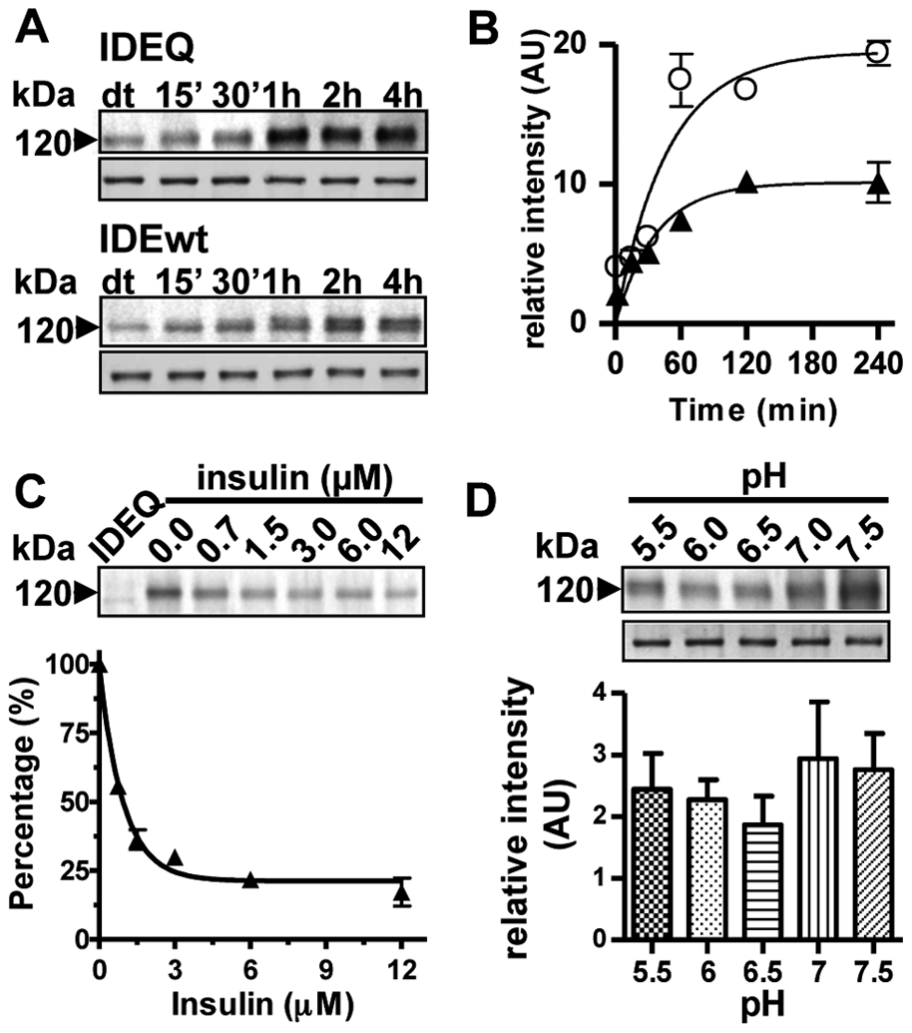
Time-course, amount and pH-dependence of the formation of complexes between Aβ1-42 and IDEwt or IDEQ. (**A**) Western blots with anti-Aβ 6E10 showing the ∼120 kDa band corresponding to IDE-AβSCx (IDE-Aβ stable complex) as a function of the incubation time. Top panel, IDEQ; lower panel, IDEwt. Both PVDF membranes were developed simultaneously with a STORM 860 scanner. Below each Western blot, the same membranes stained with Coomassie blue, show IDEwt or IDEQ loading. (**B**) Densitometric data from Western blots for IDEQ (◯) and IDEwt (▴) were fitted to a single exponential equation using Graph Pad Prism v.4 software. Points represent the mean ± SEM from two independent experiments in duplicate. (**C**) IDEQ-AβSCx formation is partially competed by pre-incubation for 1 h with insulin at the indicated molar excess before the addition of Aβ1-42. Data are expressed as the percentage of the remaining Aβ-positive band at ∼120 kDa, in arbitrary units, as a function of insulin concentration. Each point represents the mean ± SEM of two independent experiments in duplicate. Inset: a representative Western blot of IDEQ-AβSCx developed with 6E10. (**D**) Densitometry of IDEQ-AβSCx at the indicated range of pH as determined by Western blot with anti-Aβ. Bars represent the mean ± SEM of three separate experiments. Inset: top, representative Western blot with anti-Aβ of IDEQ-AβSCx; bottom, Coomassie blue of IDEQ loaded in each lane. In panels (**A**), (**C**) and (**D**), IDEwt or IDEQ-AβSCxs are indicated by arrowheads.

We first confirmed that there were no structural differences between purified IDE wild type (IDEwt) and IDEQ, as determined by static light scattering (SLS) and circular dichroism (CD) (Figure S1). The loss of enzymatic activity of IDEQ was assessed by four different methods: 1) The absence of fluorescent signal increase after co-incubation with the fluorogenic substrate V; 2) The lack of significant degradation as determined by trichloroacetic acid (TCA) precipitation of ^125^I-insulin (∼1.5% residual activity); 3) Western blots with anti-Aβ and 4) Enzyme-linked immuno-sorbent assay (ELISA) quantification of monomeric Aβ1-42 that remained measurable after 5 days of incubation with IDEQ (94.4%), IDEwt (10.4%) and IDEwt with EDTA (88.9%) at a 1∶10 molar ratio (enzyme:Aβ) (Figure S2). In a prior work [44] we showed that co-incubation of Aβ1-28 with IDEwt yielded higher levels of IDE-AβSCx when the protease was inhibited with EDTA. Thus, we tested if natively folded IDEQ produced a similar effect. A time-course of IDE-AβSCx formation was determined by quantifying the immunoreactivity of the ∼120 kDa band with anti-Aβ by Western blot. Although the half-times of IDE-AβSCx formation were similar (IDEQ = 32 min; IDEwt = 26 min), the yield of IDEQ-AβSCx was ∼3 -fold higher relative to IDEwt (Figure 1). To test whether Aβ was interacting with IDEQ substrate-binding site, as previously shown for IDEwt [44], competition was performed with insulin. Pre-incubation with increasing concentrations of insulin decreased the formation of IDEQ-AβSCx reaching a final value of ∼22% of the maximum formed in the absence of insulin (Figure 1). The fact that prevention was not complete may be attributed to several factors including the k_d_ (∼8 μM) for insulin dimerization (the size of an insulin dimer is not compatible with the volume of the IDE chamber), the non-equilibrium reaction that leads to IDEQ-AβSCx formation and that binding site may not be fully shared [44].

### IDEQ has no effect upon insulin conformation and its activity on cultured cells

**Figure 2:**
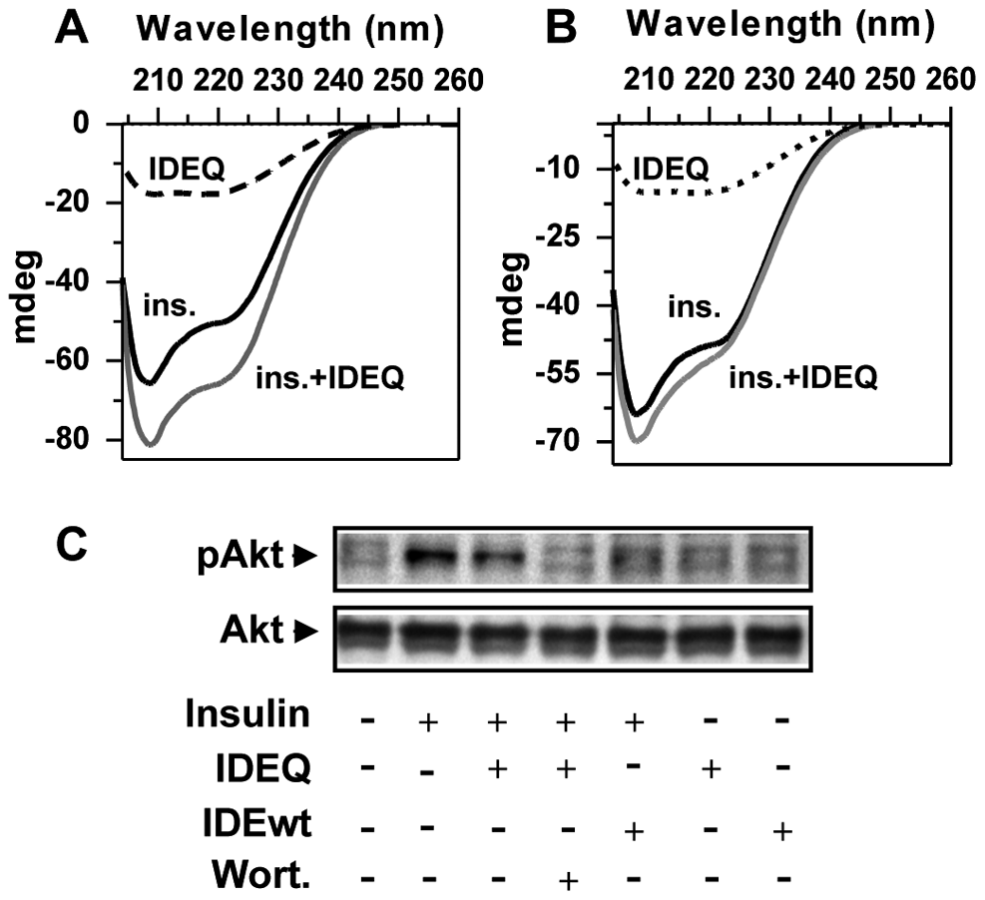
IDEQ does not modify insulin conformation. (**A**) *Circular dichroism (CD) spectra* of 10 μM insulin (ins.) in working buffer (solid black line), insulin with IDEQ at 1∶100 molar ratio, enzyme:insulin (solid gray line) and IDEQ alone (dotted line) with no prior incubation. (**B**) Same samples as in panel (**A**) after incubation for 24 h at 25°C. Insulin alone (solid black line), insulin with IDEQ (solid gray line) and IDEQ alone (dotted line). (**C**) Western blot with anti-phospho-Akt and anti-total Akt of U-87 cell lysates. Cells were exposed for 30 min with insulin alone, insulin previously co-incubated with IDEwt or IDEQ, as indicated. Wortmannin (wort) was incubated at 10 nM for 30 min before treatments.

Although insulin does not aggregate under physiological conditions and low concentrations, it has a high affinity for IDE and undergoes partial unfolding inside the IDE chamber before hydrolysis [47]. Having shown that IDEQ was natively folded and had minimal residual activity, we tested its possible effect on insulin conformation or aggregation. To address this question, two experiments were performed. Firstly, insulin was incubated alone or with IDEQ at 1∶100 (enzyme:insulin) for 24 h and secondary structure determined by CD. Signal intensities were similar and no differences were seen in the two α-helix minima of insulin with or without IDEQ at dead time and after 24 h of incubation (Figure 2). Secondly, human astrocytic U-87 cells were treated with insulin with or without pre-incubation with IDEQ. The activation of insulin receptor was assessed by quantifying Akt phosphorylation. Insulin pre-incubated with IDEwt was used as a positive control for the loss of insulin activity due to hydrolysis. Insulin treatment increased phospho-Akt levels relative to total Akt. Pre-incubation of insulin with IDEQ did not result in a significant loss of hormone activity, while pre-incubation with IDEwt abolished insulin receptor activation (Figure 2). When cells were previously treated with wortmannin, an inhibitor of PI3K, the increase in Akt phosphorylation was abrogated, indicating the specificity of insulin pathway activation in the insulin-IDEQ experiment. These results support that IDEQ, despite binding insulin, does not induce a conformational change on the hormone or promotes its aggregation.

### Inverse relationship between hydrolysis and IDE-AβSCx formation as a function of pH

The physical association between IDE and Aβ *in vivo* seems to be ubiquitous, including intracellular organelles with acidic pH [48], [49]. Therefore, we tested the effect of a range of pH in the formation of IDE-AβSCx. The activity of IDEwt was drastically reduced *in vitro* below pH 6 while the formation of IDEwt-AβSCx increased from low levels at neutral pH reaching a maximum at pH 5.5, likely related to reduced degradation of Aβ (Figure S3). Instead, IDEQ was capable of forming comparable amounts of IDE-AβSCx across the range of pH studied, as shown in Figure 1. These and our previously reported results prompted us to explore a functional significance of the stable interaction between IDE and Aβ unrelated to peptide hydrolysis.

### IDEQ reduces the size of Aβ aggregates

**Figure 3:**
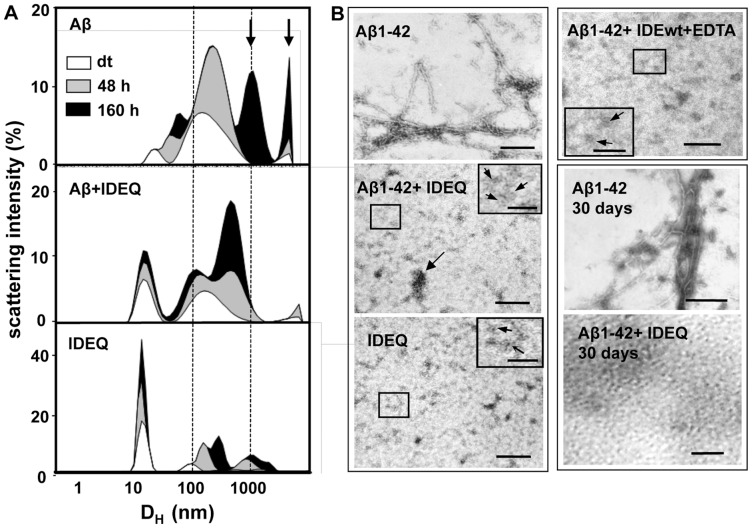
Time-course of Aβ self-assembly assessed by DLS and TEM in presence or absence of IDEQ. (**A**) Dynamic light scattering (DLS) time-course of Aβ1-42 incubated alone or in the presence of IDEQ, as indicated. White, gray and black areas correspond to “dead time”, 48 h and 160 h of incubation, respectively. Arrows indicate Aβ species with very high hydrodynamic diameter (D_H_) (≧1000 nm). Aβ1-42 was incubated at a 10-molar excess over IDEQ. Results are representative of three independent experiments. Data were plotted using Sigma Plot 5.3 software to graphically show the D_H_ (in a logarithmic scale) and the percentage of scattering intensity (I.Scatt) (in a linear scale). (**B**) Transmission electron microscopy (TEM) analysis of: Aβ1-42 alone showing typical amyloid fibrils (top left); Aβ1-42 in the presence of IDEQ (middle left) showing coalescent annular species of approximately 5–10 nm and the absence of amyloid fibrils. Arrow indicates a large aggregate. Inset, annuli indicated by small arrows; IDEQ alone (bottom left) forming annular structures of 5–10 nm (inset, large arrows) and thin short rods (inset, thin arrows); Aβ1-42 incubated with IDEwt inhibited with EDTA (top right), showing no amyloid fibril formation. Inset, arrows depict 5–10 nm annuli and rods. Electron micrographs were obtained after 6.5 days of incubation. Samples incubated for 30 days revealed typical amyloid fibrils for Aβ1-42 alone (middle right) in contrast to a homogeneous population of small annuli of 5–10 nm in the presence of IDEQ (bottom right). In all TEM experiments, molar ratios were 1∶10 (enzyme:Aβ). Bars in left panel and top and middle right panel = 100 nm, in insets, bars = 20 nm. In bottom right, bar = 30 nm.

The effect of IDEQ upon Aβ self-assembly was first studied by dynamic light scattering (DLS), which allows a rough estimate of the size of aggregates in solution. Aβ1-42 showed species with increasing hydrodynamic diameter (D_H_) and intensity of scattered light with peaks around 1000 nm after 6.5 days of incubation (Figure 3). Although the total amount of IDE in the brain is likely much higher than monomeric Aβ [50], [51], we used IDEQ at substoichiometry to minimize non-specific hydrophobic interactions *in vitro*. Aβ1-42 incubated in the presence of IDEQ at 1∶10 molar ratio (enzyme: Aβ) resulted in the detection of two distinct populations: one around 10–13 nm, as expected for IDEQ dimers, and a second population with a peak around 150 nm. Notably, only two discrete populations with D_H_ of 150 and 400 nm were seen at the end of incubation, as opposed to Aβ1-42 alone (Figure 3). Similar results were obtained using the highly amyloidogenic “Dutch” variant of Aβ peptide 1–28 in which Glu is replaced by Gln at position 22 (Aβ28Q) (not shown).

### IDEQ abolishes the formation of Aβ fibrils

To determine the morphology and size of the aggregates detected by DLS, samples were analyzed by transmission electron microscopy (TEM). After 6.5 days of incubation, Aβ1-42 (Figure 3) and Aβ28Q (Figure S4) formed typical amyloid fibrils. The effect of co-incubation with IDEQ at 1∶10 (enzyme: Aβ molar ratio) was remarkable, with a complete absence of amyloid fibril formation by Aβ1-42 or Aβ28Q. In the presence of IDEQ, much smaller structures were seen. These ranged from thin, short rods or protofibrils to coalescent annular structures with a diameter of ∼5 nm. Similar structures were seen when IDEQ was incubated alone, although the tendency to form large aggregates was higher in the Aβ-containing samples (Figure 3). When IDEwt inhibited with EDTA was incubated with Aβ1-42, non-fibrilar products were seen, reinforcing that blockage of amyloid formation was unrelated to proteolysis (Figure 3). To test if the lack of amyloid formation by Aβ in the presence of IDEQ was due to a retardation of the fibrillization pathway, samples were co-incubated for 30 days. Amyloid fibrils with the typical features were observed with Aβ1-42 alone. In contrast, only small aggregates were found in the presence of IDEQ (Figure 3). Typical amyloid fibrils were formed by Aβ28Q in the presence of bovine serum albumin (BSA) at the same molar ratio (Figure S4), indicating a specific effect of IDEQ rather than a non-specific, hydrophobic peptide-protein interaction. Overall, DLS and TEM results indicate that in the presence of IDEQ at sub-stoichiometry, Aβ forms small aggregates that lack the morphological features of amyloid fibrils. In addition, by using the shorter Aβ1-28Q, we showed that the hydrophobic transmembrane C-terminal region of Aβ was not necessary for the underlying peptide-enzyme interaction that halted amyloid formation.

### Kinetics of Aβ aggregation in the presence of IDEQ and effect of ATP

**Figure 4:**
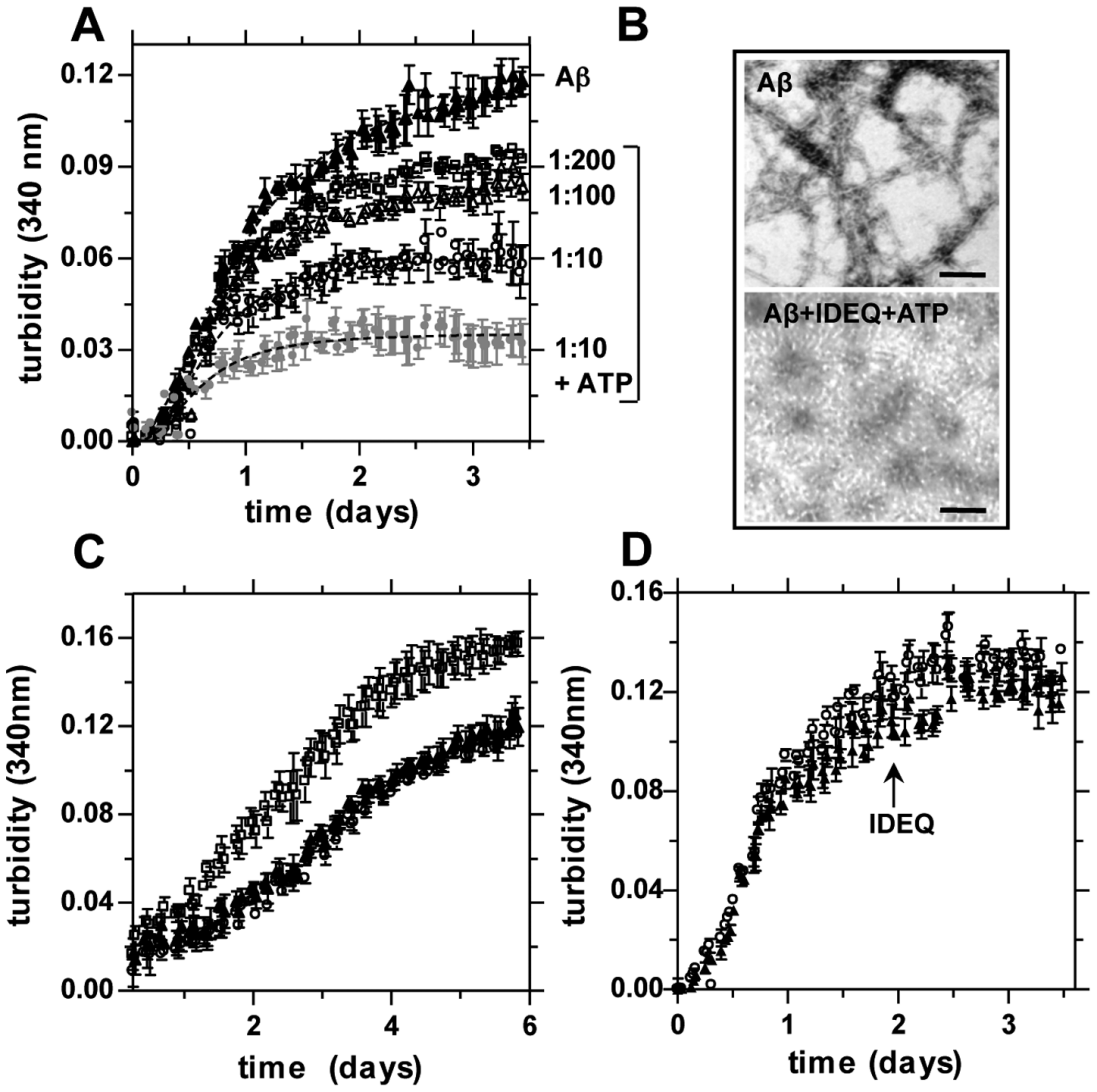
Effect of IDEQ and ATP upon the kinetic of Aβ aggregation and seeding. (**A**) Turbidity profiles of Aβ1-42 at 15 μM alone in working buffer (▴) or in the presence of IDEQ at the indicated molar ratios (IDEQ:Aβ), from top to bottom: 1∶200 (□), 1∶100 (Δ), 1∶10 (◯) and 1∶10 containing 0.5 mM ATP (**•**). Light scattering at 340 nm was measured every 30 min using a TECAN GENios multi-well reader (for clarity, only the points every other 90 min are shown). The bracket encloses the curves obtained after co-incubation of Aβ1-42 with IDEQ at the indicated conditions. Results are expressed as mean ± S.E.M. of at least two independent experiments in duplicate. (**B**) Representative TEM images of samples at steady state of Aβ1-42 alone (top) or with IDEQ at 1∶10 molar ratio in the presence of ATP (bottom). Bars = 100 nm. (**C**) Time course of Aβ1-42 aggregation alone (▴), in the presence of seeds previously formed with IDEQ (◯), or after the addition of pure Aβ1-42 seeds (□). (**D**) Kinetics of aggregation of Aβ1-42 after the addition of IDEQ (◯) or the same volume of working buffer (▴) to Aβ1-42 after 48 h of self-assembly, as indicated by the arrow.

To further characterize the effect of IDEQ on Aβ1-42 self-assembly, the kinetics of aggregation were followed by scattering at 340 nm. Aβ1-42 self-assembly showed an initial lag phase of ∼12 h, followed by exponential growth of 24–36 h until a steady state was reached after 48–72 h (the shorter periods of time for these phases as compared with the rest of the experimental conditions was due to the repeated agitation every 30 min, as described in Materials and Methods). Typical amyloid fibrils were obtained at steady state, as assessed by TEM (Figure 4). At a molar ratio of 1∶10 (enzyme: Aβ), IDEQ slowed the rate of aggregation and the scattered intensity. This effect was concentration-dependent and it could still be observed at a substoichiometry (IDEQ: Aβ) of 1∶200. Interestingly, co-incubation with ATP, which binds IDE and modulates its conformation and catalytic activity upon short substrates [52], [53], showed an increase in the anti-aggregation effect (Figure 4). Such enhancement was independent of ATP hydrolysis as confirmed by using the non-hydrolysable ATP-PNP (data not shown). Moreover, it could not be attributable to an increase in the minimal residual proteolytic activity of IDEQ (Figure S2). To further characterize the products in the presence of ATP, these were visualized by TEM at steady state. A homogeneous population of protofibrils of ∼10 nm was seen in the Aβ samples co-incubated with IDEQ and ATP in contrast to the typical amyloid fibrils formed by Aβ alone (Figure 4). The incubation of IDEQ and Aβ at a 1∶1 stoichiometry did not yield a lower absorbance than IDEQ-Aβ at 1∶10 with ATP (data not shown). However, this result was not unexpected in light of the rather large aggregates seen under TEM.

### IDEQ cannot dissociate Aβ fibrils and Aβ aggregates formed in its presence are incompetent for seeding

Aβ1-42 was incubated alone for 48 h and then fresh IDEQ was added at a 1∶10 molar ratio. Turbidity measurements after IDEQ addition were similar to those of Aβ1-42 alone, suggesting that no disaggregation took place and that IDEQ was not capable of dissociating amyloid fibrils (Figure 4). We next addressed whether Aβ aggregates formed in the presence of IDEQ were competent to seed the self-assembly of monomeric Aβ. An aliquot of Aβ1-42 aggregated in the presence of IDEQ (at 1∶10 molar ratio, enzyme:peptide) was taken at steady state, sonicated and added to a fresh Aβ1-42 solution. As expected, seeds obtained by sonication of fibrillar Aβ1-42 shortened the lag phase and increased the light scattering plateau. Strikingly, the “seeds” obtained from the co-incubation of Aβ1-42 with IDEQ had no effect on the aggregation rate when compared with Aβ1-42 self-assembly alone, as shown by the super-imposable scattering curves in both conditions (Figure 4). When end-products obtained from steady state were analyzed by TEM, typical amyloid fibrils were observed in case of Aβ1-42 with or without seeds, yet it tended to be longer in the former (not shown). These results suggest that IDEQ not only modifies the kinetics of Aβ self-assembly but that non-fibrillar Aβ species formed in the presence of IDEQ lack the ability to act as templates for monomeric Aβ to follow the amyloidogenic pathway.

### Differential solubility and secondary structure of Aβ species formed in the presence of IDEQ

**Figure 5:**
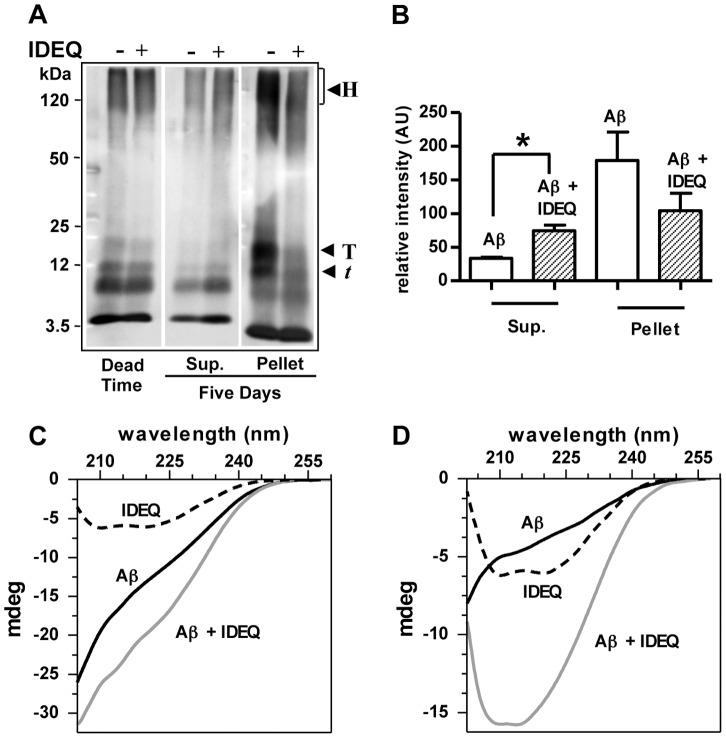
Effect of IDEQ upon solubility and secondary structure of Aβ aggregates. (**A**) samples containing Aβ1-42 before incubation (“dead time”) and after incubation for 5 days with or without IDEQ were centrifuged at 3,000× g for 5 min and supernatants and pellets analyzed by Western blots with anti-Aβ 6E10 and 4G8. Arrowheads indicate: H, high molecular mass oligomers, T, Aβ tetramers and *t*, Aβ trimers. (**B**) Densitometric quantification of Aβ1-42 obtained from Western blots shown in panel (**A**). Bars represent the mean ± SEM of total Aβ immunoreactivity in arbitrary units (AU). * p<0.05, Student's *t* test. (**C**) Far UV-CD spectra recorded at “dead time” of Aβ1-42 (solid black line), IDEQ alone (dotted line) and Aβ1-42 co-incubated with IDEQ (solid gray line) at a 1∶300 molar ratio (IDEQ:Aβ). (**D**) Far UV-spectra recorded after 6 days of incubation of Aβ1-42 alone or Aβ1-42 with IDEQ at 1∶300 molar ratio. Samples were centrifuged as described above and supernatants analyzed. IDEQ alone, dotted line; Aβ1-42 alone, solid black line; Aβ1-42 co-incubated with IDEQ, solid gray line.

To study the effect of IDEQ upon Aβ1-42 solubility, samples were co-incubated for 5 days, centrifuged at low speed and analyzed by Western blot. Total immunoreactivity of Aβ1-42 in the supernatant was 2-fold higher after incubation with IDEQ as compared to Aβ1-42 alone. Accordingly, a higher proportion of Aβ1-42 partitioned to the pellet in the absence of IDEQ (Figure 5). Major differences in Aβ pellets were accounted for by trimers, tetramers and high molecular mass SDS-resistant oligomers. To determine the secondary structure of soluble Aβ oligomers formed alone or in the presence of IDEQ, Aβ1-42 was incubated at 1∶300 molar ratio (to minimize enzyme signal) and far UV-CD spectra analyzed. The effect of IDEQ in preventing amyloid fibril formation at this molar ratio was assessed by TEM (Figure S5). IDEQ alone showed a spectrum with minima at 208 and 222 nm, consistent with its reported structure [46]. In contrast, the spectra of fresh Aβ1-42 alone or Aβ1-42 with IDEQ were mainly unstructured, in accordance with the negligible contribution of signal by IDEQ (Figure 5). After 6 days of incubation, samples were centrifuged to separate large aggregates and far UV-CD spectra obtained from the supernatants. Soluble Aβ1-42 in the absence of IDEQ showed a minimum at ∼213 nm, but the bulk of the peptide was unstructured with one third of its original intensity, consistent with the results obtained by Western blots (Figure 5). Notably, Aβ1-42-IDEQ spectrum was at least 2-fold more intense than Aβ1-42 alone displaying minima at 208 and 214 nm. These results suggest that soluble Aβ1-42 species formed in the presence of IDEQ, either bound or unbound to the enzyme, were more structured than soluble Aβ1-42 incubated without IDEQ.

### Aβ1-42 aggregates formed in the presence of IDEQ are not neurotoxic

**Figure 6:**
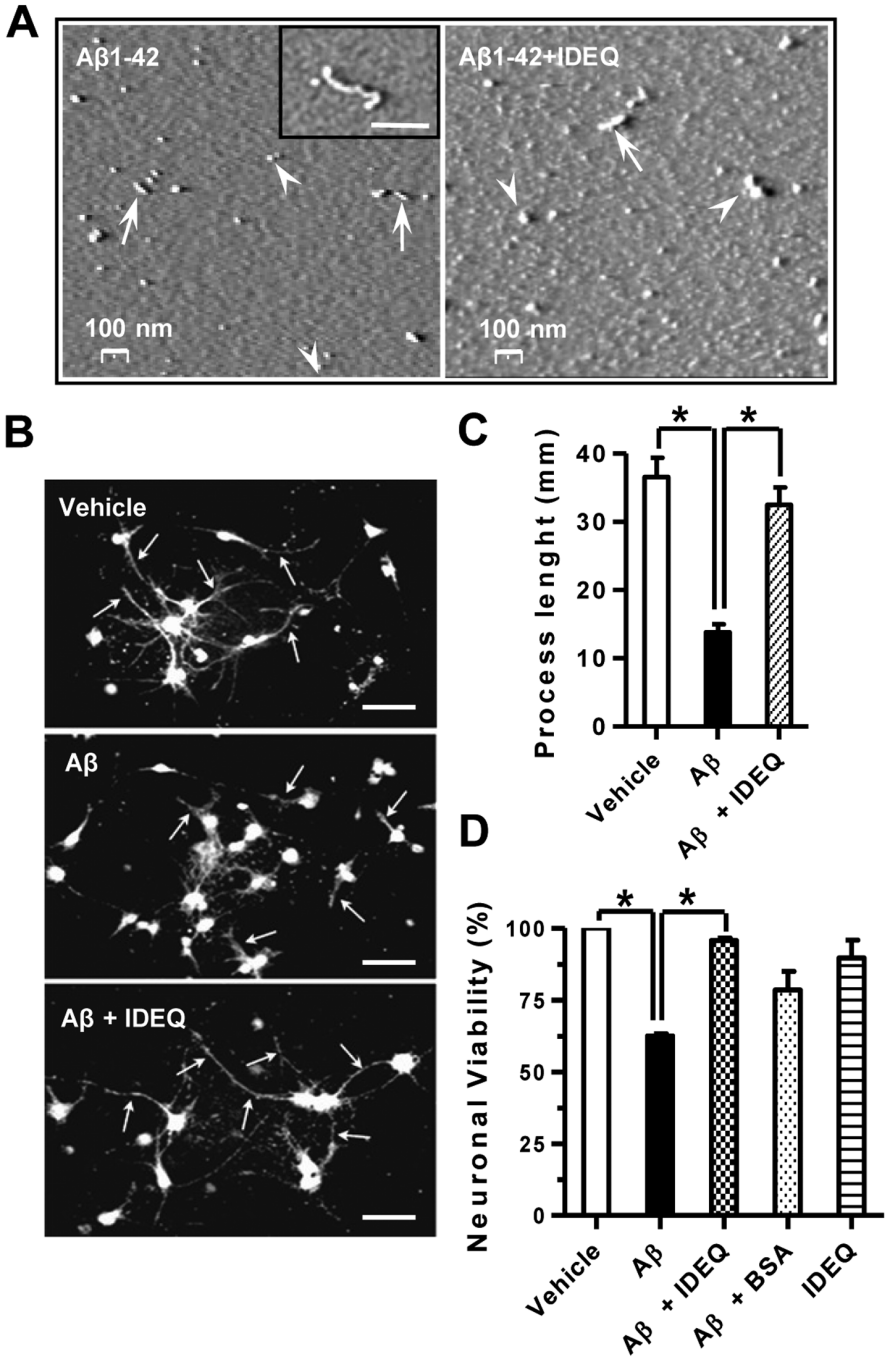
Aβ1-42 oligomers formed in the presence of IDEQ are not neurotoxic. (**A**) Representative AFM images showing the size and morphology of Aβ1-42 neurotoxic species. Left, Aβ1-42 incubated alone for 4 days. Approximately spherical species of ∼20–30 nm are indicated by arrowheads. Rods and short protofibrils are depicted by arrows. Inset: a larger Aβ1-42 protofibril is shown. Right; Aβ1-42 incubated in the presence of IDEQ at a 1∶10 molar ratio (IDEQ: Aβ) showing larger aggregates of 50–60 nm (arrowheads) and rods with lengths of ∼100–120 nm (arrows). (**B**) Representative immunofluorescence of primary differentiated neurons exposed to vehicle, Aβ1-42 alone or Aβ1-42 pre-incubated with IDEQ from top to bottom, as indicated. White arrows point at neuronal processes. Bars  = 30 μM. (**C**) Analysis of neuronal processes under the conditions as shown in panel (**A**). Bars represent the mean ± SEM of processes' lengths as measured from the centre of the neuronal body * p<0.01, one-way ANOVA, Tukey post-hoc test. (**D**) Viability of mature primary neurons after the indicated treatments as assessed by MTT reduction. * p<0.05, one-way ANOVA, Tukey post-hoc test. Results are shown for three independent experiments.

The Aβ aggregates seen by DLS and TEM together with the CD data raised the possibility that co-incubation with IDEQ resulted in the generation of toxic Aβ non-fibrillar species. When Aβ was incubated alone for 4 days, mainly spherical oligomers, rods and protofibrils were seen under atomic force microscopy (AFM) (Figure 6). In the presence of IDEQ, aggregates were more heterogeneous and slightly larger as compared to Aβ alone (Figure 6). Primary neurons exposed to Aβ1-42 aggregates showed a remarkable retraction and nicking of their processes and a ∼40% reduction in viability. In contrast, cells exposed to Aβ1-42 aggregates obtained from co-incubation with IDEQ had no morphological alterations and were ∼95% viable as compared with control cells (Figure 6). Co-incubation of Aβ1-42 with BSA showed a trend toward lower toxicity although it was not statistically different from that induced by Aβ1-42. To rule out a possible trophic effect upon neuronal viability of IDEQ unrelated to its effect on Aβ1-42 aggregation, cells were exposed to IDEQ alone and showed no significant differences with vehicle treatment.

## Discussion

The catalytically inactive IDEQ was capable of forming a highly stable complex with Aβ1-42 more efficiently than IDEwt. In agreement with our prior work using IDEwt, competition with insulin suggested that at least part of the substrate binding site was involved [44]. IDEQ-AβSCx was generated in a broad range of pH, including that compatible with cellular secretory and endocytic vesicles in which both IDE and Aβ are present *in vivo* [49], [54]. These properties of IDEQ suggested another inherent function of IDEwt unrelated to hydrolysis. Purified IDEwt aggregates at pH<6 after long incubation times required for full Aβ self-assembly and retains a ∼10% of activity. Thus, to rule out hydrolysis-dependent effects we used the inactive mutant IDEQ. It is important to remark that the ELISA assay is not capable of detecting Aβ in a stable complex with IDEQ. Therefore, the residual activity of IDEQ (∼6%) as determined by this method, is likely overestimated.

At neutral pH, the size and morphology of Aβ aggregates were drastically modified by IDEQ. Aβ aggregates were smaller and did not form amyloid fibrils in the presence of IDEQ, as shown by DLS and TEM. Instead, small annuli, rods and protofibrils were found, even after 30 days of co-incubation, ruling out retardation or a purely kinetic effect upon Aβ self-assembly. The time-course of turbidity experiments showed that 1) IDEQ at substoichiometry down to 1∶200 was sufficient to slow down the lag and exponential phases with a lower turbidity at steady-state; 2) the addition of IDEQ at the end of the exponential growth phase was ineffective to prevent amyloid formation or to modify its kinetics; 3) sonicated end-products of Aβ-IDEQ co-incubation were incompetent to induce the “seeding” of Aβ monomers, indicating that they did not form amyloidogenic templates. These results are consistent with IDEQ exerting its effect at early stages of Aβ aggregation, most likely before the exponential phase of amyloid fibril elongation. Interestingly, the presence of ATP at its k_d_ enhanced the anti-amyloidogenic effect of IDEQ. This effect was independent of ATP hydrolysis and may be related with the reported stimulation of the catalytic activity of IDEwt upon very short peptide substrates by polyphosphate anions (see below). Western blots of insoluble and soluble aggregates showed a 2-fold increase of Aβ species in the supernatant in the presence of IDEQ, including intermediate and high molecular mass SDS-resistant oligomers with a likely content of β structure. The potential neurotoxicity of these Aβ oligomers and aggregates was ruled out by the exposure of primary neuronal cultures in which Aβ, in the presence of IDEQ, yielded non-toxic species as assessed by neuronal viability and morphology.

Regarding the possible mechanism underlying the anti-amyloidogenic effect of IDEQ, we propose that it relies on a chaperone-like activity. Similar effects upon Aβ aggregation have been described for Hsps 70/40, Hsp90, Hsp104 and small heat shock proteins (sHsp20) [30], [31], [55]. These effects include the blockage of the amyloid pathway resulting in non-fibrillar aggregates, the competence at substoichiometry, and their activity at early stages of the aggregation process, except for Hsp104 which shows activity at different stages [55]. In some cases, Aβ oligomers formed in the presence of chaperones have been proven to be non-toxic to neuronal cell lines [31].

The three-dimensional structure of IDEQ crystallized with several substrates [46] including insulin and Aβ1-40 displays features that are relevant to the interpretation of our findings: 1) the presence of a chamber that has the approximate volume of a native insulin monomer, enclosed by the amino-terminal half (domains 1 and 2) and the carboxyl-terminal half (domains 3 and 4); 2) the existence of a substrate binding “exosite” (∼30 Å away from the catalytic residues) that contributes to guide the partial unfolding of substrates; 3) the stabilization of short β strands common to all substrates (not present in the free state in solution) at the “exosite” and at the catalytic site; 4) the alternation between closed and open states of the IDE chamber articulated upon a flexible loop connecting IDE halves which may be the rate limiting step for the catalytic cycle; 5) an inner acidic surface of domains 3 and 4 that contributes to stabilize the “closed” conformation in the absence of substrate and 6) the relatively slow *Kcat* (in the order of min ^−1^) for amyloidogenic peptides [56], [57].

Our initial observation of an IDEQ-Aβ highly stable complex with an apparent 1∶1 stoichiometry (after SDS treatment) and occupying at least part of the binding sites led us to speculate that after binding, a population of Aβ alone or together with a partner sequence on IDE may provide a template for additional binding of Aβ monomers or small oligomers. IDE-Aβ complexes may then progress to form non-amyloid aggregates. Although the closed IDEQ chamber may be limiting in this mechanism, the binding of at least another Aβ monomer would impose a steric hindrance for “IDE closure”, facilitating the assembly process upon the IDEQ-Aβ template. The major polyanion binding site has been recently proposed by crystallographic analysis to be located at a positively charged inner surface contributed by IDE domains 3 and 4 [53]. The enhancement of the anti-amyloidogenic effect by ATP may be related with a reduced electrostatic attraction between IDE halves, favoring the open state and the aggregation of Aβ upon IDEQ-Aβ templates. We propose that such aggregation takes place much faster (∼30 min) than the lag phase of amyloidogenic Aβ self-assembly and lowers free monomer concentration, a limiting factor for nuclei formation.

Although insulin is non-amyloidogenic under physiological conditions, the high affinity between IDE and insulin, the assisted partial unfolding of insulin inside the IDE chamber and the lack of proteolysis by IDEQ, raised the question whether IDEQ could induce stable insulin conformational changes and/or self-aggregation. Notably, despite a long co-incubation, IDEQ had no effect upon insulin conformation as assessed by CD and receptor activation on cultured cells.

Taken together, these results are consistent with IDEQ having a chaperone-like activity upon Aβ aggregation with a holdase or mixed (“foldase-holdase”) type of mechanism. Proteases and chaperones share the capability of binding partially folded or unfolded polypeptides. Both activities have been extensively studied in proteins of the C1pP, FtsH and Lon families; highly conserved ATP-dependent proteases with chaperone activity [58], [59]. Proteases in which the catalytic site is required for a chaperone-like activity include the aspartic proteases pepsin and HIV protease, as shown by following the aggregation of denatured citrate synthase or rhodanese [60]. In the same work, thermolysin -a typical M13 Zn^2+^ metallopeptidase- did not show chaperone activity but this remains to be tested with relatively short peptides such as Aβ. In gram-negative bacteria, the IDE ortholog, pitrilysin (PTR) is targeted to the periplasmic space in which it is believed to degrade small peptides derived from leader sequences [61]. In eukaryotes, IDE has been found in cytosol, plasma membrane, endocytic pathway, mitochondria, exosomes and extracellular space [42]. Several cellular factors may impact upon IDE proteolytic activity. Acidic pH, oxygen reactive species, oxidized glutathione and nitric oxide can reduce the catalytic function of IDE [62], [63]. Moreover, it has been shown that naturally occurring IDE missense mutations in a rat model of DM2 reduce IDE proteolytic activity upon insulin and Aβ [64]. Therefore, under physiological (low pH in endocytic and exocytic vesicles) or pathological conditions (oxidative stress, inflammation, mutations), a latent chaperone activity of IDE may become relevant to prevent Aβ toxic aggregation.

The M16 family members presequence protease (PreP) in chloroplasts and human metalloprotease 1 (MP1) in mitochondria are capable of degrading Aβ [65], [66], [67]. PreP crystal structure and its proposed mechanism of recognition and catalysis are similar to those of PTR and IDE, including a “peptidasome” chamber that encloses peptide substrates [68]. A chaperone-like activity of IDE and related M16 proteases such as PTR or PreP and MP1 under suboptimal conditions for hydrolysis may enhance their efficiency in preventing the self-assembly of small peptides into toxic oligomers. By driving the formation of non-toxic and more soluble aggregates, such activity may facilitate their cellular disposal by general degradation systems.

## Materials and Methods

### Site-directed mutagenesis

Rat IDE cDNA 42–1059 was kindly provided by Dr. Richard Roth, Stanford University). The Glu by Gln substitution at position 111 was introduced by PCR using the forward primer: 5′CCTGGCTTAAGCCATTTTTGTCAGCATATGCTGTT-TTTGGGAACA3′ and the complementary reverse primer. After cloning in plasmid pET 30 a (+) (Novagen), whole plasmid was amplified and the PCR product treated with Dnp I. Digestion of the methylated or hemi-methylated DNA template allowed the selection of the mutation-containing DNA. To confirm that the desired mutation was correctly introduced, the IDE insert was sequenced using a primer for the T7 promoter.

### Expression and purification of IDEwt and IDEQ

IDEwt and IDEQ were expressed in E. coli BL21 transformed with pET 30 a (+) vector containing the corresponding cDNAs. Bacteria were cultured in TB medium containing 100 μg/mL kanamicin at 37°C and protein expression induced with 0.5 mM isopropyl-β-D- 1-thiogalactopyranoside (IPTG). The first purification step was done with a His-Trap Ni^2+^ affinity chromatography (GE Healthcare) in 50 mM Tris-HCl, pH 8.0, 300 mM NaCl followed by elution with a linear gradient from 20 to 250 mM imidazole in the same buffer. The second purification step was size-exclusion chromatography (SEC) with a Superdex 200 columm (Amersham) in 50 mM NaCl, 20 mM Tris-HCl, pH 7.4 (working buffer). After SEC, protein purity was determined by SDS-PAGE and quantified by measuring absorbance at 280 nm using the IDE extinction coefficient (115810 M^−1^ cm^−1^). Concentration was expressed as monomeric IDE.

### Aβ peptides preparation and IDE-AβSCx formation

Aβ1-40 (Sigma) and Aβ1-42 (American Peptide Co) were acquired as lyophilized powder. Aβ1-28Q was kindly provided by Jorge Ghiso (New York University). Peptides were first dissolved in 1,1,1,3,3,3-hexa-fluoro-2-propanol (HFIP) at 1 mg/ml, incubated at room temperature for 8 h and stored at −80°C until use. To disaggregate possible Aβ nuclei, prior to each experiment an aliquot was taken from the stock solution and sonicated for 10 min on ice. After removing HFIP with gentle streaming of nitrogen, peptides were dissolved in working buffer and sonicated for 10 min on ice. Finally, peptides were centrifuged for 30 min at 10,000× g to pellet insoluble material and the supernatants filtered through 0.22 μm spin filters (Sigma). To obtain IDEwt or IDEQ AβSCx, purified enzymes and peptide were incubated at 1 μM and 35 μM, respectively, in working buffer at 25°C for the indicated times. At the end of incubation, samples were analyzed by Western blots as described below. Data were fitted to a single exponential equation to calculate half-lives as described [44].

### IDE activity assays

IDEwt and IDEQ enzymatic activities were assessed by four different methods. 1) Hydrolysis of the fluorogenic peptide 7-methoxycoumarin-RPPGFSAFK-2, 4-dinitrophenyl (“substrate V”, R&D Systems) containing the bradykinin sequence. The enzymatic activity of IDEwt (10 nM) was determined using increasing concentrations of substrate V in working buffer, pH 7, for 10 min at 37°C. The sigmoidal plot of V_0_ as a function of substrate concentration allowed us to calculate a K_0.5_ ∼12.5 μM. To compare the activities of IDEwt and IDEQ, proteins were incubated at 10 nM with substrate V at 10 μM in working buffer at 37°C for up to 10 min. The increase in fluorescence was monitored with an Aminco-Bowman spectrofluorometer at 320 nm and 405 nm as excitation and emission wavelengths, respectively. 2) TCA precipitation of porcine insulin degradation. Briefly, 20,000 cpm of ^125^I-insulin (specific activity 300 μCi/μg, kindly provided by Edgardo Poskus, University of Buenos Aires) were incubated for 30 min at 37°C in working buffer containing 0.25% BSA and 10 ng of IDEwt or IDEQ. ATP was added at 0.5 mM to IDEQ, as indicated. The reaction was stopped by addition of 0.1 ml of 15% TCA. Samples were centrifuged at 12,000× g for 30 min at 4°C and c.p.m. determined in pellets and supernatants using an automatic gamma-counter. 3) Western blots after incubation of Aβ1-42 with or without IDEQ were performed as described below. 4) ELISA quantification was performed on samples with Aβ1-42 (15 μM) alone or in the presence of IDEwt, IDEwt containing 5 mM EDTA or IDEQ at 1∶10 molar ratios (enzymes: peptides). At “dead-time” or after 5 days of incubation at room temperature, aliquots of 2 μl were mixed with 2 μl of distilled water and 1 μl of 5M guanidine-HCl in water. Aβ42 levels were quantified by using a commercial ELISA kit following the manufacturer's instructions (Invitrogen).

### IDEwt activity assays in pH range 5.5 to 7.5

The effect of pH on IDEwt activity was determined in a pH range from 5.5 to 7.5 using the fluorogenic peptide substrate V as described above. One hundred μM citric acid or 100 μM PO_4_NaH_2_/ PO_4_Na_2_H were used to reach the specified pH in each case.

### Circular dichroism and thermal denaturation of IDEwt and IDEQ

Far UV-CD spectra measurements were carried out in a Jasco J-815 spectropolarimeter equipped with a Peltier temperature-controlled sample holder device. Spectra of IDEwt or IDEQ at 0.5 μM in working buffer at 25°C were acquired before and after thermal denaturation at 1°C/min using a 0.2 cm path length quartz cuvette. Final spectra were obtained after the subtraction of background readings of working buffer. Thermal denaturation was followed by plotting the signal at 222 nm.

### Static light scattering assays (SLS)

The average molecular masses of IDEwt or IDE-Q were determined with a Precision Detector PD2010 light scattering instrument. Briefly, 200 μg of a purified batch of IDEwt or IDEQ were injected into a Superdex G-200 column (Amersham) connected to an HPLC system Bromma LKB 2248 and eluted in working buffer at 0.5 ml/min. To estimate the average molecular mass of proteins, the 90° static light scattering signal and UV absorbance or refractive index of the eluting material were recorded and analyzed with the Discovery 32 software (Precision Detectors). The 90° light scattering detector was calibrated using bovine serum albumin (66.5 kDa) as a standard. Average values obtained from four independent measurements of the apparent molecular masses of IDE dimers and tetramers were ∼250 and ∼500 kDa, respectively.

### Dynamic light scattering (DLS)

Aβ monomeric species, obtained as described above, were incubated at 50 μM in working buffer at 25°C for the indicated times with or without IDEQ at a molar ratio 1∶10 (enzyme:peptide). The same molar ratio was used for BSA as a control for specificity. Particle size measurement was assessed using the 633 nm excitation laser and detector at 173° with a Zetasizer Nano-S (Malvern Instruments). Results were expressed as the intensity of scattered light as a function of D_H_ and time of incubation.

### Transmission electron microscopy (TEM)

Aliquots were taken from the same samples used for DLS measurements or from samples at steady state of turbidity experiments (see below). Ten μl were adsorbed for 1 min onto formvar-coated nickel grids (Electron Microscopy Science) and stained with 1% uranyl acetate aqueous solution for 1 min. Grids were visualized on a Zeiss 10C Transmission Electron Microscope.

### Turbidity assessment of Aβ aggregation kinetics and seeding

One hundred and fifty μl of Aβ1-42 at 10 μM were loaded per well in a 96-multiwell polystyrene plate with flat bottoms (Nunc). Co-incubation with IDEQ was done at the indicated molar ratios. Plates were placed in a TECAN Genios multi-well reader and peptide aggregation followed by measuring absorbance at 340 nm every 30 min with a 10-s mixing shake before each measure. Pre-formed seeds were obtained from samples at steady state by sonication for 5 min on ice. The seeding assay was carried out by adding 1% v/v of seeds to a fresh preparation of monomeric Aβ. All experiments were performed at constant 25°C.

### AFM characterization of non-fibrillar Aβ1-42 species

Aliquots of Aβ1-42 or Aβ1-42 with IDEQ were collected after 4 and 6 days of incubation, deposited onto freshly cleaved mica and left for 1 min at room temperature. The specimens were then rinsed with deionized water and dried with a nitrogen stream (1 bar). Images were collected after dynamic scanning force microscopy in air by using a Veeco Explorer AFM fitted with a tube-type 2 micrometer scanner and antimony doped silicon cantilever tips (spring constant 1–5 N/m; Veeco). Tapping/Non-contact mode using a FESP-MT tip and a gain of 2.0 V were used. Three sample regions covering 4 μm^2^ were randomly documented and 100 to 1500 elements from each region were analyzed. Particles' heights, widths and lengths were measured by using the Nanotec WSxM 4.0 Image Browser software.

### CD assessment of Aβ and insulin-IDEQ co-incubation

Aβ1-42 at 15 μM in working buffer alone or in the presence of IDEQ at 1∶300 molar ratio (enzyme:peptide) were incubated for 6 days at 25°C. Spectra were acquired at dead-time and at the end of incubation over a wavelength range of 260–202 nm using a Jasco J-815 spectropolarimeter in 10-mm path cuvettes. Data interval was set at 0.2 nm and the average of five scans was obtained for each experimental conditions. Insulin secondary structure was determined in the presence or absence of IDEQ at a 1∶100 molar ratio (enzyme:peptide) incubated for 24 h at 25°C. In both experiments, UV-CD spectra were smoothed with an algorithm provided by JASCO after the subtraction of working buffer background signal.

### SDS-PAGE and Western Blots

Proteins were subjected to electrophoresis on 7.5 or 10% polyacrylamide SDS-Tris-tricine gels, stained with Coomassie blue or transferred to PVDF membranes (GE Healthcare). For Western blots, membranes were blocked with 5% low fat milk in PBS containing 0.1% Tween for 2 h at room temperature and incubated with the indicated primary antibodies overnight at 4°C. Anti-Aβ monoclonal 6E10 and 4G8 were obtained from Signet. Anti-rabbit or anti-mouse horseradish peroxidase-labeled antibodies were used for detection using ECL Plus reagent (Amersham Biosciences). Enhanced chemiluminescence signals were scanned with a STORM 860 fluorometer and analyzed with ImageQuant 5.1 software (Molecular Dynamics). To quantify IDE-AβSCx formation, the amount of total IDE in each lane was estimated by Coomassie blue staining of the same PVDF membranes used for fluorescence detection.

### Primary neuronal cultures

Neuronal cultures were obtained from rat embryos as described [69]. Briefly, hippocampi were dissected free of meninges, suspended in 0.5 ml of Ca^2+^ and Mg^2+^ – free Hank's balanced solution and treated with 0.25% trypsin (Invitrogen) for 25 min at 37°C. Immediately after trypsinization, tissue was washed with Neurobasal medium (Gibco) containing 10% fetal bovine serum supplemented with B-27 and N-2, 50 U/ml penicillin and 50 mg/ml streptomycin (NBSM). A cellular suspension was obtained by using fire-polished Pasteur pipettes. For immunofluorescence, isolated cells were plated at 60/mm^2^ on 12-mm coverslips. For viability assessment, cells were plated at 3×10^4^ on P-96 wells. After 3 h of incubation under 5% CO_2_ at 37°C to permit cell attachment, medium was replaced with fresh medium without serum. Neuronal cultures with a purity of 90–95% were obtained. Cultures were maintained in NBSM without serum for 14 days to allow neuronal differentiation.

### Inmunofluorescence and neurotoxicity assay

To assess neuronal damage induced by Aβ1-42, cells were examined by immunofluorescence (IF). The remaining viability was determined using an (3-(4,5-Dimethylthiazol-2-yl)-2,5-diphenyltetrazolium bromide (MTT) assay. Neurons were exposed for 20 h to 10 μM Aβ1-42 in NBS, washed with PBS and fixed with 4% paraformaldehyde. For immunofluorescence, cells were permeabilized with 0.1% Triton X-100 in PBS and blocked with PBS containing 0.1% BSA. To recognize specifically neuronal morphology, anti-β III tubulin monoclonal IgG (Sigma) was incubated for 1 h, followed by Cy3-labeled goat anti-mouse IgG. Nuclei were labelled with the blue-fluorescent Hoechst acid stain. Images were captured using an Olympus BX60 microscope with a DP Controler 3.1.1.267 PC program. The average length of neuronal processes was determined using Image J software. Quantitative estimation of remaining cellular viability was assessed by the reduction of MTT by mitochondria of living cells. MTT was incubated at 0.5 mg/ml for 2 h at 37°C and products of reaction dissolved in 0.1N HCl and 2% SDS in water. Absorbance was measured at 570 nm and viability expressed as the O.D. relative to neurons treated with vehicle alone. As a positive control for neuronal damage, cells were exposed to 0.05% H_2_O_2_ (not shown). Quantitative data of three independent MTT assays were analyzed by one-way ANOVA and a post-hoc Tukey test with Graph Pad Prism v. 4.0. All results represent the mean ± SEM and p<0.05 was considered statistically significant.

### Insulin receptor activation assay

Human glioma U-87 cells (ATCC HBT-14) were cultured at 37°C with 5% CO_2_ in DMEM high glucose media supplemented with non-essential amino acids (PPA) and 2 mM piruvic acid. Cells at 80% confluence were deprived of serum for 24 h and exposed to 100 nM insulin for 30 min. Insulin was co-incubated with IDEQ or IDEwt for 24 h at 25°C before cellular exposure. After treatments, cells were washed with cold PBS, harvested and lysed by passage through a 25-gauge needle 20 times in 150 mM PO_4_H_2_Na, pH 7.5 containing 150 mM NaCl, 0.5% deoxycholic acid, 1% Triton X-100 and a cocktail of protease inhibitors (Sigma). Homogenates were centrifuged for 10 min at 12,000× g and protein concentration in the supernatant determined with a Pierce BCA kit. To detect Akt and phosphorylated Akt at Ser 473 under different conditions, 65 μg of total protein were separated on 10% SDS-PAGE and transferred to PDVF membranes. Polyclonal rabbit anti-Akt and monoclonal mouse anti-p-Akt antibodies from Cell Signaling were used for Western blots. To assess the specificity of signaling, phophatidyl-inositol-3 kinase (PI3K) was inhibited by incubating cells with 100 nM wortmannin (Millipore) for 30 min prior to insulin exposure.

### Ethics Statement

The protocol for the care, handling and use of animals followed the ARRIVE guidelines [70] and was approved by the Fundación Instituto Leloir Institutional Animal Care and Use Committee.

## Supporting Information

Figure S1 **Purity and conformation of IDEQ.** (**A**) Coomassie blue staining of a 7.5% SDS-PAGE showing the purity of recombinant IDEwt and IDEQ obtained from SEC. Two μg of IDEwt or IDEQ were loaded in each lane. On the left, molecular mass markers are indicated in kilodaltons (kDa). (**B**) Thermal denaturation of IDEwt (◯) and IDEQ (•) at 0.8 μM was assessed by monitoring signal loss at 222 nm as a function of increasing temperature. Proteins were incubated in working buffer (20 mM Tris-HCl, pH 7.4, containing 50 mM NaCl) at 37°C for 5 min before the initiation of a temperature increase from 25°C to 85°C at 1°C per min. Inset, far UV CD spectra of proteins before (IDEQ, black solid line; IDEwt, gray solid line) or after thermal denaturation (IDEQ, black dotted line; IDEwt, gray dotted line). (**C**) Elution profiles obtained for IDEwt and IDEQ after SEC with a Sephadex G-200 column showing the molecular masses of native IDEwt and IDEQ dimers (D), solid arrows) and tetramers (T), dashed arrows) by SLS arranged in a flow-cell fashion.
(TIF)

Figure S2 **Loss of proteolytic activity of IDEQ.** (**A**) IDEwt initial velocity of degradation (V_0_) as a function of substrate V concentration. K_50_  = 12.5 μM. (**B**) Time course of the hydrolysis of fluorogenic substrate V at 10 μM in the presence of IDEwt (◯) or IDEQ (□). Fluorescence in the absence of IDE (buffer alone) is depicted by (⧫). (**C**) IDE activity as assessed by insulin degradation. ^125^I-insulin was incubated for 30 min at 37°C in working buffer containing 0.25% BSA and 10 ng IDEwt or IDEQ (with or without ATP) followed by precipitation with trichloroacetic acid (TCA). Results are expressed as ^125^I-insulin remaining in the pellets. Bars represent the mean ± SEM of two independent experiments performed in duplicate. (**D**) Top, representative Western blot showing the lack of degradation of Aβ1-42 by IDEQ in the presence of 0.5 mM ATP. Aβ1-42 was incubated alone or with IDEQ (1∶10 molar ratio, enzyme:Aβ) at 25°C for 2 h and separated on a 10% SDS-PAGE. Bottom, Coomassie blue staining of the same membrane used for Western blot showing IDEQ (arrow). (**E**) ELISA measurement of Aβ1-42 after 5 days of incubation with, IDEQ, IDEwt or IDEwt in the presence of EDTA, as indicated. Results are expressed as the percentage of monomeric Aβ1-42 referred to Aβ levels in non-incubated samples as 100%. Bars represent the mean ± SEM of two independent experiments. In all cases, molar ratios were 1∶10 (enzymes:Aβ).
(TIF)

Figure S3 **Activity and IDE-AβSCx formation as a function of pH.** (**A**) IDEwt activity was measured using substrate V at 10 μM for 5 min at 37°C and expressed as the percentage of activity relative to pH 7.5. Bars represent the mean ± SEM of three independent measurements. (**B**) Top, IDEwt-AβSCx formation (arrowhead) as determined by Western blot with 6E10. The same membrane was stained with Coomassie blue to control for IDEwt loading, as shown below the Western blot. Bottom, densitometric quantification. Bars represent the mean ± SEM in arbitrary units (A.U.) of IDE-AβSCx immunoreactivity.
(TIF)

Figure S4 **TEM analysis of the effect of IDEQ upon amyloid formation by Aβ28Q.** (**A**) Aβ28Q alone showing typical amyloid fibrils. (**B**) Aβ28Q co-incubated with IDEQ showing large coalescent aggregates (arrow) of annular structures of less than 10 nm (inset, large arrow) and short protofibrils (thin arrow). (**C**) Aβ28Q incubated with BSA showing the formation of amyloid fibrils. Bars = 100 nm; insets, bars = 20 nm; H, bar = 30 nm.
(TIF)

Figure S5 **TEM analysis of the IDEQ-Aβ preparation used for CD measurements.** (**A**) Aβ1-42 after 6 days of incubation showing typical amyloid fibrils. Bar = 100 nm. (**B**) Aβ1-42 incubated with IDEQ at a 1∶300 molar ratio (enzyme: Aβ) for 6 days. Short arrows indicate annuli or 5–10 nm, long arrows indicate short rods or protofibrils ∼5 nm wide and 15–30 nm long. Bar = 30 nm.
(TIF)
