# Supplementary figures and images for: Proteolytically Inactive Insulin-Degrading Enzyme Inhibits Amyloid Formation Yielding Non-Neurotoxic Aβ Peptide Aggregates
Source: PLoS One. 2013 Apr 11;8(4):e59113. doi: 10.1371/journal.pone.0059113 (PMC3623905; doi:10.1371/journal.pone.0059113)

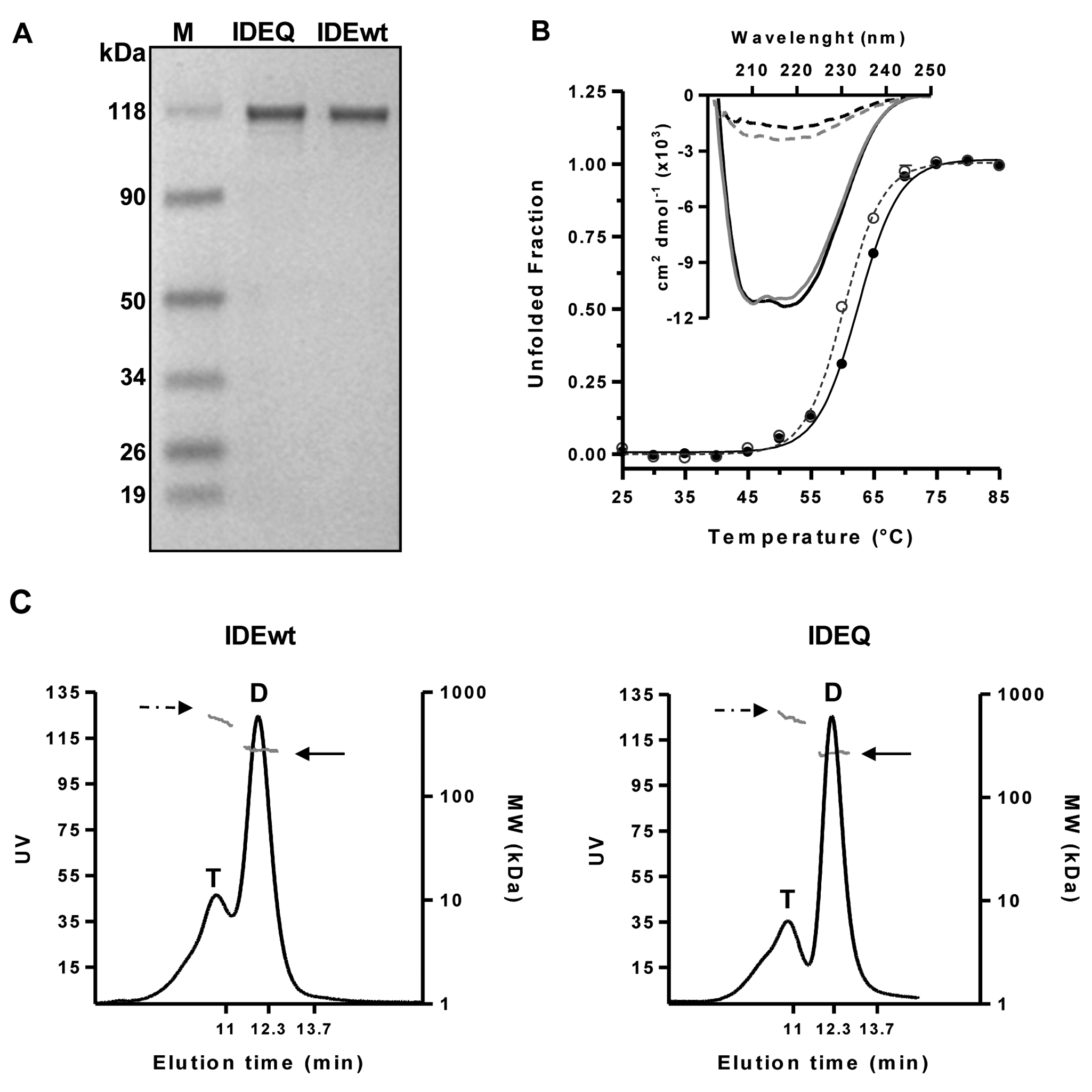

Supplement: Figure S1 — Purity and conformation of IDEQ. (A) Coomassie blue staining of a 7.5% SDS-PAGE showing the purity of recombinant IDEwt and IDEQ obtained from SEC. Two μg of IDEwt or IDEQ were loaded in each lane. On the left, molecular mass markers are indicated in kilodaltons (kDa). (B) Thermal denaturation of IDEwt (◯) and IDEQ (•) at 0.8 μM was assessed by monitoring signal loss at 222 nm as a function of increasing temperature. Proteins were incubated in working buffer (20 mM Tris-HCl, pH 7.4, containing 50 mM NaCl) at 37°C for 5 min before the initiation of a temperature increase from 25°C to 85°C at 1°C per min. Inset, far UV CD spectra of proteins before (IDEQ, black solid line; IDEwt, gray solid line) or after thermal denaturation (IDEQ, black dotted line; IDEwt, gray dotted line). (C) Elution profiles obtained for IDEwt and IDEQ after SEC with a Sephadex G-200 column showing the molecular masses of native IDEwt and IDEQ dimers (D), solid arrows) and tetramers (T), dashed arrows) by SLS arranged in a flow-cell fashion. (TIF) [file pone.0059113.s001.tif]

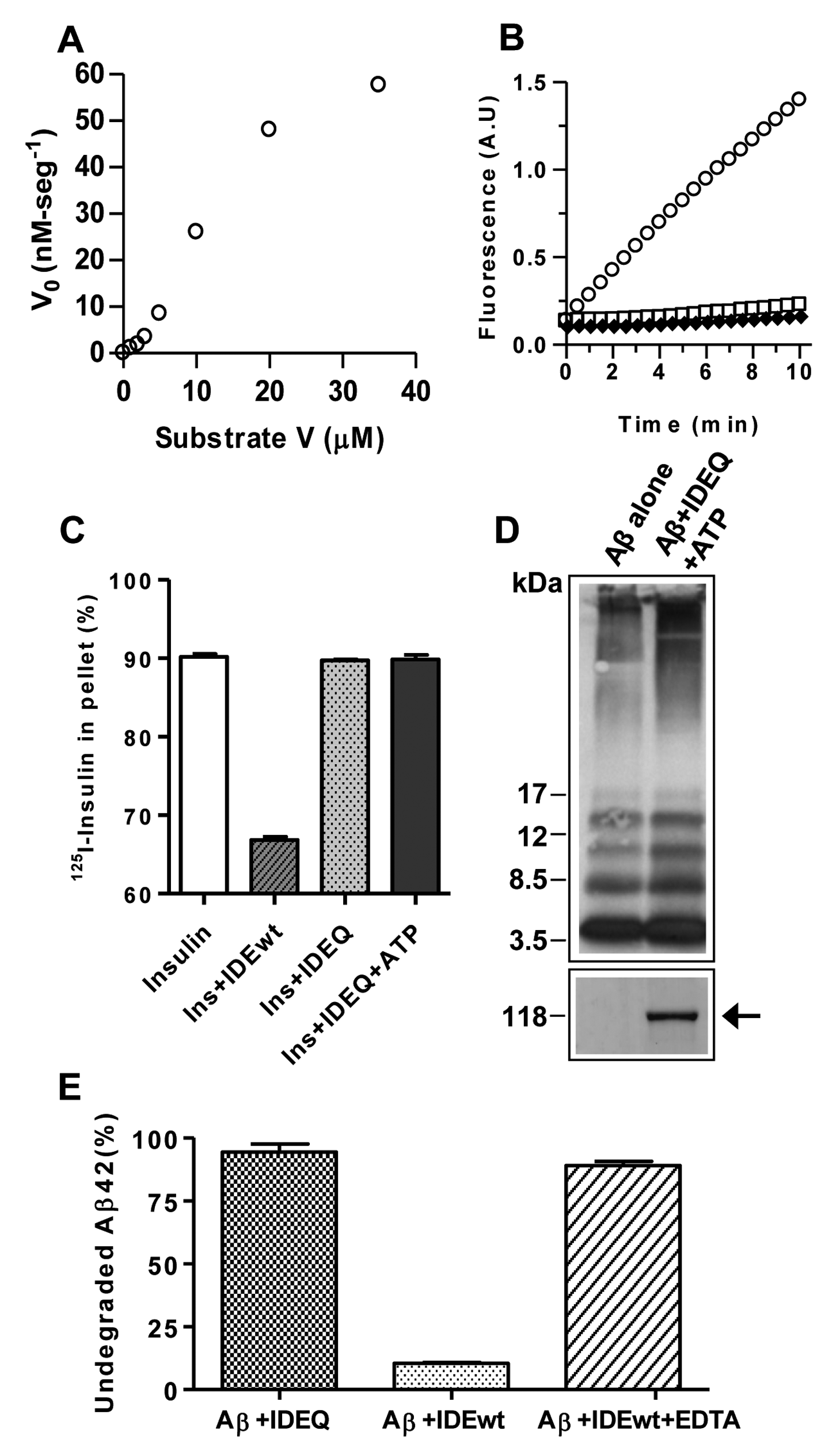

Supplement: Figure S2 — Loss of proteolytic activity of IDEQ. (A) IDEwt initial velocity of degradation (V0) as a function of substrate V concentration. K50 = 12.5 μM. (B) Time course of the hydrolysis of fluorogenic substrate V at 10 μM in the presence of IDEwt (◯) or IDEQ (□). Fluorescence in the absence of IDE (buffer alone) is depicted by (⧫). (C) IDE activity as assessed by insulin degradation. 125I-insulin was incubated for 30 min at 37°C in working buffer containing 0.25% BSA and 10 ng IDEwt or IDEQ (with or without ATP) followed by precipitation with trichloroacetic acid (TCA). Results are expressed as 125I-insulin remaining in the pellets. Bars represent the mean ± SEM of two independent experiments performed in duplicate. (D) Top, representative Western blot showing the lack of degradation of Aβ1-42 by IDEQ in the presence of 0.5 mM ATP. Aβ1-42 was incubated alone or with IDEQ (1∶10 molar ratio, enzyme:Aβ) at 25°C for 2 h and separated on a 10% SDS-PAGE. Bottom, Coomassie blue staining of the same membrane used for Western blot showing IDEQ (arrow). (E) ELISA measurement of Aβ1-42 after 5 days of incubation with, IDEQ, IDEwt or IDEwt in the presence of EDTA, as indicated. Results are expressed as the percentage of monomeric Aβ1-42 referred to Aβ levels in non-incubated samples as 100%. Bars represent the mean ± SEM of two independent experiments. In all cases, molar ratios were 1∶10 (enzymes:Aβ). (TIF) [file pone.0059113.s002.tif]

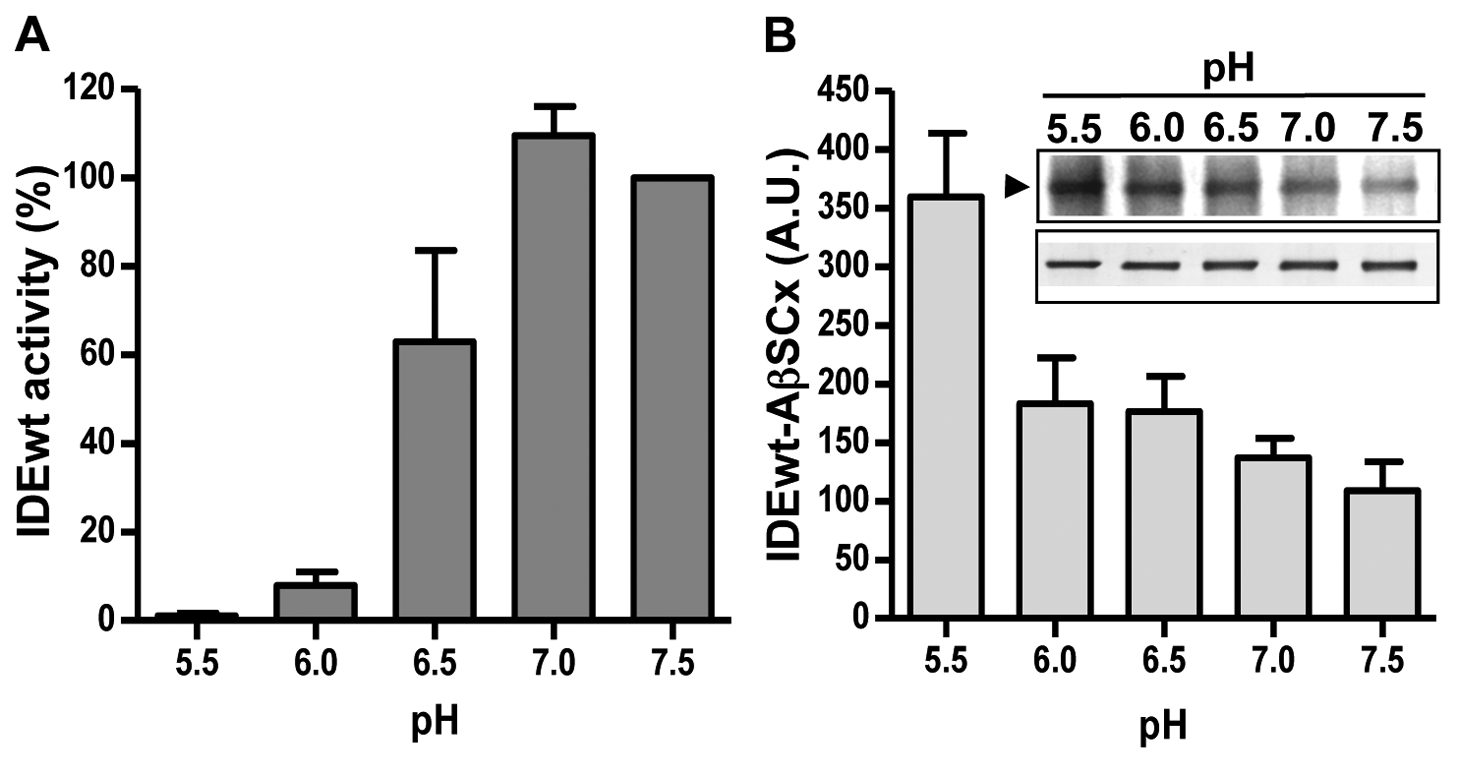

Supplement: Figure S3 — Activity and IDE-AβSCx formation as a function of pH. (A) IDEwt activity was measured using substrate V at 10 μM for 5 min at 37°C and expressed as the percentage of activity relative to pH 7.5. Bars represent the mean ± SEM of three independent measurements. (B) Top, IDEwt-AβSCx formation (arrowhead) as determined by Western blot with 6E10. The same membrane was stained with Coomassie blue to control for IDEwt loading, as shown below the Western blot. Bottom, densitometric quantification. Bars represent the mean ± SEM in arbitrary units (A.U.) of IDE-AβSCx immunoreactivity. (TIF) [file pone.0059113.s003.tif]

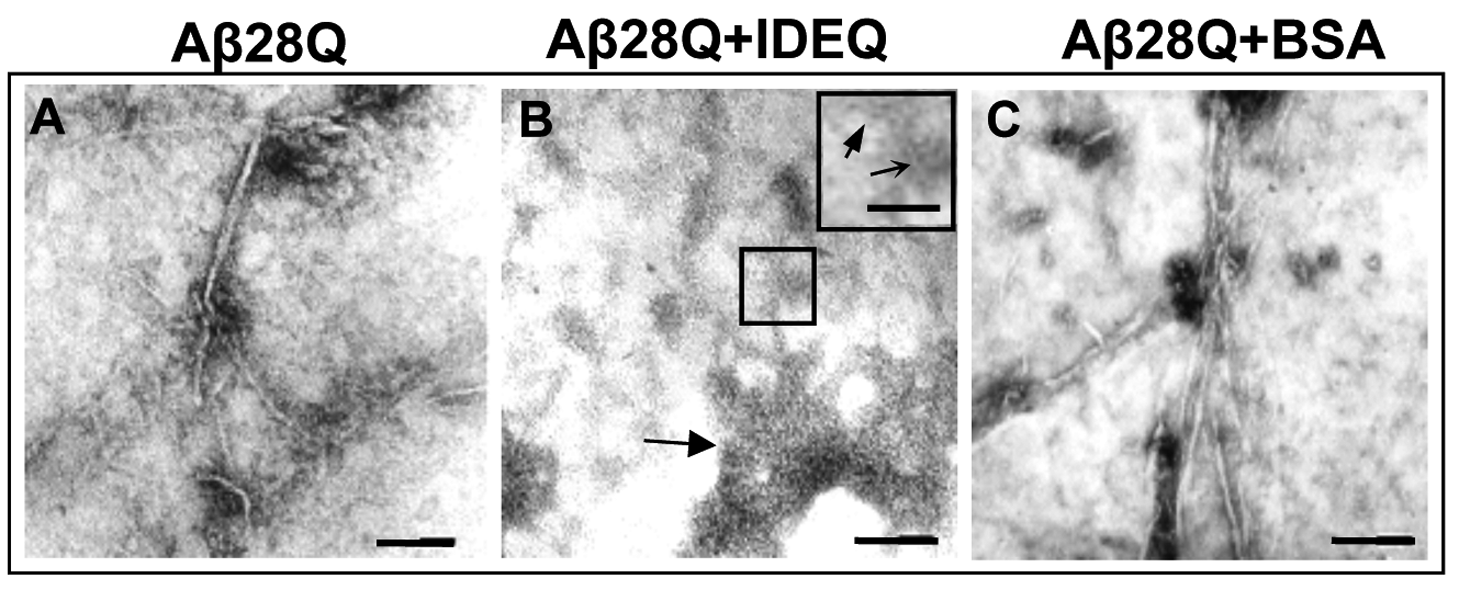

Supplement: Figure S4 — TEM analysis of the effect of IDEQ upon amyloid formation by Aβ28Q. (A) Aβ28Q alone showing typical amyloid fibrils. (B) Aβ28Q co-incubated with IDEQ showing large coalescent aggregates (arrow) of annular structures of less than 10 nm (inset, large arrow) and short protofibrils (thin arrow). (C) Aβ28Q incubated with BSA showing the formation of amyloid fibrils. Bars = 100 nm; insets, bars = 20 nm; H, bar = 30 nm. (TIF) [file pone.0059113.s004.tif]

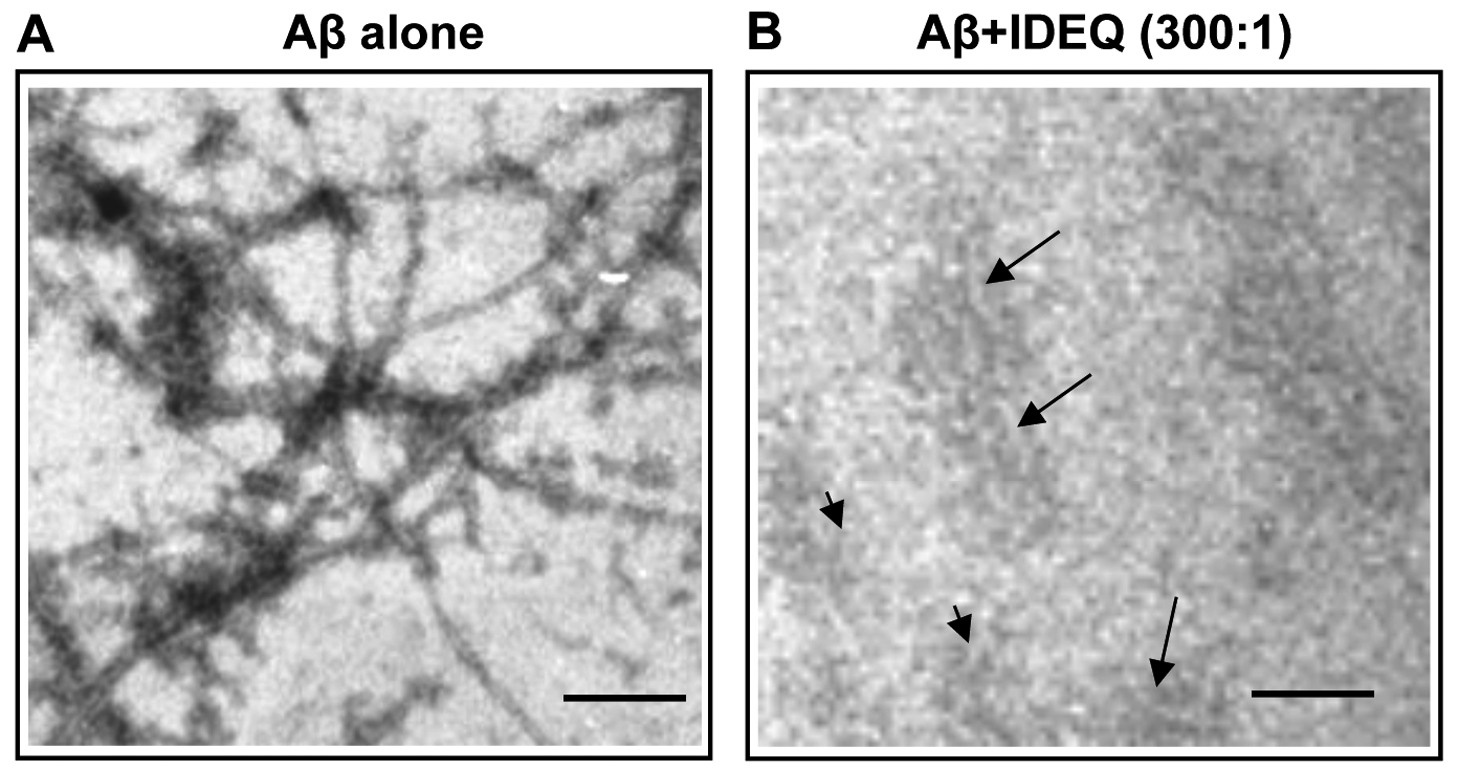

Supplement: Figure S5 — TEM analysis of the IDEQ-Aβ preparation used for CD measurements. (A) Aβ1-42 after 6 days of incubation showing typical amyloid fibrils. Bar = 100 nm. (B) Aβ1-42 incubated with IDEQ at a 1∶300 molar ratio (enzyme: Aβ) for 6 days. Short arrows indicate annuli or 5–10 nm, long arrows indicate short rods or protofibrils ∼5 nm wide and 15–30 nm long. Bar = 30 nm. (TIF) [file pone.0059113.s005.tif]
